# Unilateral spontaneous rupture of a testicular implant thirteen years after bilateral insertion: a case report

**DOI:** 10.1186/1752-1947-4-341

**Published:** 2010-10-26

**Authors:** Michael St J Floyd, Helen Williams, Sanjay K Agarwal, Alan R De Bolla

**Affiliations:** 1 Dept of Urology, Wrexham Maelor Hospital, Croesnewydd Road, Wrexham, LL13 7TD, UK; 2 Department of Radiology, Wrexham Maelor Hospital, Croesnewydd Road, Wrexham, LL13 7TD, UK

## Abstract

**Introduction:**

We describe a case of spontaneous, non traumatic rupture of a single artificial testis in a patient who had undergone bilateral, staged radical orchidectomy followed by prosthesis insertion. The consequences and radiological appearances of implant rupture are discussed. We believe it is the longest time interval recorded between prosthesis insertion and rupture.

**Case presentation:**

A 50 year old Caucasian man presented to our outpatient department with an altered consistency in his right testicular prosthesis without any systemic symptoms or local inflammation. His left testicular prosthesis had retained its consistency since insertion.

**Conclusion:**

The majority of cases reported to date have required exploration due to symptoms but we describe a case that was managed conservatively.

## Introduction

Prosthesis insertion is commonplace following radical orchidectomy as it provides patients with a cosmetically normal scrotum. The first case of a prosthetic testis was described in 1941 by Girdansky and Newman using a Vitallium implant [1]. Puranik in 1973 [2] in the paediatric population and Lattimer in 1973 [3] in adults are credited with introducing a silicone gel filled implant that resembled a naturally feeling testis. Implants consist of an outer silicone elastomer which envelops a transparent gel. Complications with breast implants have been well documented and include pain, deformity and autoimmune phenomenon. Following concerns over silicone breast implants the American Urological Association in 1992 advised against the use of silicone gel testicular implants and advocated the use of silicone elastomer prostheses instead [4].

Specific to urological use implants can extrude by shedding of the outer elastomer shell or via direct leakage of the gel. Other complications include scrotal contraction, migration into the inguinal canal, infection, pain, and rarely haematoma [5]. Immune complications such as human adjuvant disease have also been documented [6]. However, unlike breast implants testicular prostheses enjoy an environment that allows greater mobility, less friction, decreased vascularity and a more favourable temperature.

## Case Presentation

A 50 year old man presented to our outpatient department with a three month history of an altered consistency in his right testicular prosthesis. There was no history of trauma, pain or systemic upset. Scrotal examination revealed a palpable left testicular prosthesis and an irregular soft mass was noted in right hemiscrotum. The overlying skin was normal and no regional adenopathy was evident.

His past history was remarkable for a right testicular teratoma seventeen years earlier treated by radical orchidectomy and adjuvant chemotherapy (Belomycin, Etoposide and Carboplatin). Twelve months following this he underwent retroperitoneal lymph node dissection for residual adenopathy. Four years later he represented with a second testicular tumour in his left testis which was treated with radical orchidectomy. Histology revealed malignant teratoma which was again treated with adjuvant chemotherapy. Following his second radical orchidectomy he opted for bilateral testicular prosthesis insertion in 1996 with concomitant testosterone replacement therapy. Follow up since insertion had been unremarkable.

**Figure 1:**
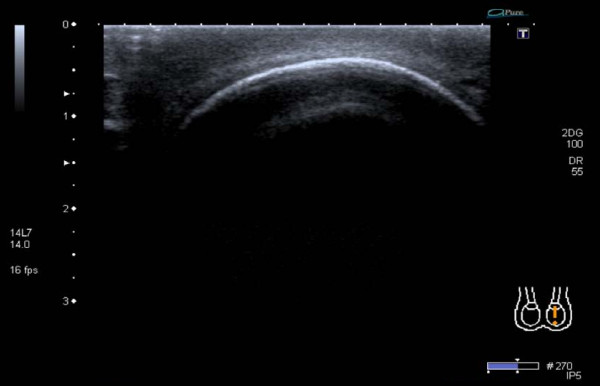
**Longitudinal section of the left side of the scrotum showing an intact prosthesis**.

**Figure 2:**
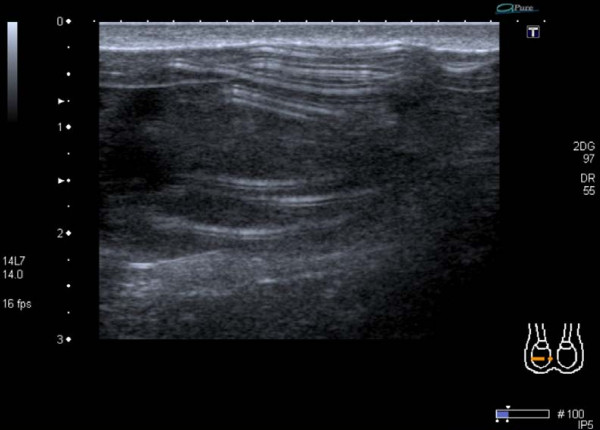
**Horizontal sections of the right side of the scrotum showing reverberation artefact in a "stepladder" sign from a ruptured prosthesis shell**.

Preliminary laboratory investigations revealed normal full blood count, renal profile, erythrocyte sedimentation rate and tumour markers. Scrotal ultrasonography revealed a normal contralateral left testicular prosthesis (figure 1) and a ruptured right prosthesis with reverberation artefact described as a "stepladder" pattern [7] on sonographic findings typically found in breast prosthesis rupture (figure 2). Following discussion with the patient, and in view of his asymptomatic state it was decided to leave the prosthesis *in situ *and adopt a conservative management strategy with biannual outpatient review.

Rupture remains an infrequent occurrence [8]. It is accepted that the longer the time interval between initial native testis removal and placement of a prosthesis the greater the incidence of complication [5]. John* et al *have previously documented a twelve year interval between placement and rupture in a patient who required exploration and prosthesis removal [9]. In this case the patient had noticed no difficulties with his bilateral implants up to thirteen years post insertion. Hage* et al *in 1999 described cases of unilateral testicular implant rupture in a selected series of patients who had undergone transgender surgery with concomitant neoscrotal formation and bilateral implants. All of these patients had a history of trauma or suspected intraoperative puncture and all underwent exploration of the affected area [10].

## Conclusions

Although we describe a unilateral rupture in a patient who had two prosthetic testes our case differs as implantation had occurred following orchidectomy for neoplasia. Additionally, our patient displayed no signs of locoregional disease and there was no history of trauma. Finally, we opted to manage this spontaneous rupture conservatively thus avoiding exploration thirteen years after insertion.

## Consent

Written consent was obtained from the patient for publication of this case report and accompanying images. A copy of the written consent is available for review by the Editor-in-Chief of this journal.

## Competing interests

The authors declare that they have no competing interests.

## Authors' contributions

MSJF identified the case as educationally important, acquired all relevant clinical data and wrote the initial and final version. HW performed the literature search and assisted in the writing. SKA performed all the radiology and interpreted the images for publication. ARDB supervised the writing and edited the final version. All authors read and approved the final manuscript.
